# A Prospective Evaluation of Duplex Ultrasound for Thoracic Outlet Syndrome in High-Performance Musicians Playing Bowed String Instruments

**DOI:** 10.3390/diagnostics8010011

**Published:** 2018-01-25

**Authors:** Garret Adam, Kevin Wang, Christopher J. Demaree, Jenny S. Jiang, Mathew Cheung, Carlos F. Bechara, Peter H. Lin

**Affiliations:** 1 Michael E. DeBakey Department of Surgery, Baylor College of Medicine, Houston, TX 77030, USA; 2 Department of Medicine, Keck School of Medicine, University of Southern California, Los Angeles, CA 90007, USA; 3 Department of Medicine, Tulane University School of Medicine, New Orleans, LA 70112, USA; 4 Department of Medicine, University of Texas MD Anderson Cancer Center, Houston, TX 77030, USA; 5 Univesity Vascular Associates, Los Angeles, CA 90024, USA; 6 Department of Surgery, Stritch School of Medicine, Loyola University Chicago, Maywood, IL 60153, USA

**Keywords:** thoracic outlet syndrome, nerve entrapment syndrome, musician, bowed string instrument, violin, viola, cello

## Abstract

Thoracic outlet syndrome (TOS) is a neurovascular condition involving the upper extremity, which is known to occur in individuals who perform chronic repetitive upper extremity activities. We prospectively evaluate the incidence of TOS in high-performance musicians who played bowed string musicians. Sixty-four high-performance string instrument musicians from orchestras and professional musical bands were included in the study. Fifty-two healthy volunteers formed an age-matched control group. Bilateral upper extremity duplex scanning for subclavian vessel compression was performed in all subjects. Provocative maneuvers including Elevated Arm Stress Test (EAST) and Upper Limb Tension Test (ULTT) were performed. Abnormal ultrasound finding is defined by greater than 50% subclavian vessel compression with arm abduction, diminished venous waveforms, or arterial photoplethysmography (PPG) tracing with arm abduction. Bowed string instruments performed by musicians in our study included violin (41%), viola (33%), and cello (27%). Positive EAST or ULTT test in the musician group and control group were 44%, and 3%, respectively (*p =* 0.03). Abnormal ultrasound scan with vascular compression was detected in 69% of musicians, in contrast to 15% of control subjects (*p =* 0.03). TOS is a common phenomenon among high-performance bowed string instrumentalists. Musicians who perform bowed string instruments should be aware of this condition and its associated musculoskeletal symptoms.

## 1. Introduction

Thoracic outlet syndrome (TOS) is due to the compression of the neurovascular structure in the thoracic inlet, as it commonly affects individuals who perform repetitive upper extremity physical activity. Neurogenic TOS, which is the most common subtype of TOS, often represents a diagnostic challenge for clinicians due in part to a lack of definitive imaging modality as well as non-specific neuromuscular symptoms which may include pain, paresthesia, weakness, fatigue, or tingling sensation of the affected arm. The diagnosis of TOS primarily requires a comprehensive assessment of clinical symptoms as well as physical examination findings. Pertinent findings on the physical examination in patients with TOS can include positive Elevated Arm Stress Test (EAST) and Upper Limb Tension Test (ULTT). Clinical studies have also highlighted an important diagnostic value of duplex ultrasound and photoplethysmography (PPG) which can detect vascular compression with dampened waveform in patients with TOS [1,2].

Individuals who participate in high-level repetitive physical activity involving the upper extremity have an increased risk of developing TOS. Previous reports have linked TOS to high-performance athletes who engaged in repetitive overhead motions, such as baseball pitchers, volleyball players, and swimmers [3,4,5]. High-performance musicians who play instruments with repetitive arm motions are at risk of developing similar musculoskeletal ailments and nerve entrapment syndromes commonly seen in athletes, with typical examples including carpal tunnel syndrome, ulnar neuropathy, and cubital tunnel syndrome [6,7,8,9]. However, studies regarding thoracic outlet syndrome in high-performance musicians remain scarce. We recently reported a case series of professional violinist and violaists who underwent successful first rib resection and scalenectomy for neurogenic TOS [10]. We hypothesize that professional bowed string musicians, who perform musical instruments with repetitive arm motions, have an increased incidence of TOS. In this prospective study, we analyzed possible thoracic outlet syndrome using both physical examinations and ultrasound assessments in high-performance bowed string musicians. 

## 2. Materials and Methods

This was a prospective study of TOS of 64 professional or elite bowed string musicians who were recruited on a volunteer basis from three metropolitan symphony orchestra, two collegiate symphony orchestra, and four professional rock bands. Fifty-two healthy non-musician subjects were also recruited which formed the control group. Inclusion criteria included subjects with a full range of motion of the upper extremities who did not suffer from any musculoskeletal ailments which limited their arm mobility. Exclusion criteria included those with a history of upper extremity injury or orthopedic procedures with movement limitations. Consent was obtained from all participants, and the study was conducted with the approval of institutional review board. The project identification code is IRB# 43529975BYC. The date of approval is 3 March 2012.

Each participant completed a questionnaire related to upper extremity activities in his or her daily routine. Associated symptoms with upper extremity activities were surveyed. Each participant underwent a detailed upper extremity physical exanimation by a board certified vascular surgeon including palpation over scalene triangle and subcoracoid space for localized tenderness. Provocative maneuvers including EAST and ULTT were also performed, and techniques for these provocative maneuvers were based on a previous publication [11]. 

Duplex ultrasound was performed in each subject by certified vascular ultrasonographers, in which subclavian artery and vein with their respective velocities were recorded. Measurements for each limb were obtained with the head turned 90 degree to abducted contralateral arm. Abnormal scans were defined as ipsilateral compression with greater than 50% increase or decrease in velocity in the subclavian artery or vein on abduction compared to adduction. A portable ultrasound unit SonoSite Edge II (Fujifilm SonoSite, Inc. Bothell, WA, USA) with an 8–5 MHz bandwidth transducer probe was placed in the infraclavicular space, with the vessel imaged in both longitudinal as well as perpendicular fashion to obtain a round cross-sectional image. In all arm positions, the waveforms were recorded as phasic, bidirectional, continuous, minimally continuous, or absent. Images and waveforms were compared between the two groups. 

Physiologic assessment of arterial flow variations based on arm position was performed. A PPG sensor was placed in the subject’s index finger of the extremity under study in which the corresponding PPG tracings were analyzed using a non-invasive vascular system Flo-Lab 2100-SX2 (Park Medical Electronics, Inc., Aloha, OR, USA). PPG waveforms were interpreted as normal, dampened, or absent. In an identical fashion, arterial flow velocities were obtained at rest and in all positions. Velocities in both resting and provocative positions were recorded and analyzed. The velocity ratio of provocative position to resting position was calculated. 

Data were expressed as mean ± standard deviation. Statistical analysis was performed using Fisher’s exact test or Pearson’s chi-square test in categorical variables. Wilcoxon rank-sum test was used to test for differences in continuous variables. All statistical analysis were performed using a statistical software program (SAS Institute, Cary, NC, USA). Statistical significance was accepted with a *p-*value of less than 0.05.

## 3. Results

Among the bowed string musicians, there were 26 violinists (41%), 21 violaists (33%), and 14 cellists (27%). No difference is noted with regards to their age and gender distribution between the musician and control groups, which were shown in Table 1. The musician group reported a significantly longer daily repetitive upper extremity activity with a mean of 5.3 ± 2.4 h, which was in contrast to 0.6 ± 0.4 h of daily activity in the control group (*p =* 0.001). The musicians reported playing musical instruments while control subjects noted computer activity were their respective reasons for daily repetitive upper extremity activity.

Results of physical examination with regards to localized tenderness on palpation in the scalene triangle and subcoracoid space were displayed in Table 2. Twenty-two musicians (34%) experienced localized tenderness to palpation in the scalene triangle, in contrast to one control subject (2%) with localized scalene triangle tenderness. Greater proportion of these musicians showed a left sided scalene triangle tenderness compared to right (25% vs. 9%, *p =* 0.04). Three musicians (5%) and one control subject (2%) developed subcoracoid space tenderness (NS). Combining the results of scalene triangle and subcoracoid space tenderness, the musician group had a greater positive exam for localized tenderness compared to the control group (39% vs. 4%, *p =* 0.01). Comparison of provocative maneuvers between the two groups were shown in Table 3. Eighteen musicians (28%) had positive EAST and 17 musicians (27%) had positive ULTT in response to provocative maneuvers, in contrast to two control subjects (4%) to each of these maneuvers (*p =* 0.04). A greater tendency of left arm positive EAST or ULTT than right arm is observed in the musician group. Combining the results of EAST and ULTT assessments, the overall positive provocative maneuvers in the musician and control group were 44% and 6%, respectively (*p =* 0.03).

Subclavian vessel compression based on ultrasound assessment in the musician group and control groups revealed 47% versus 12%, respectively (Table 4, *p =* 0.04). The laterality of subclavian vessel compression in these groups were shown in Table 4. Among the musicians, there is a greater subclavian vessel compression in the left arm compared to the right side (36% vs. 22%). No difference in laterality of subclavian vessel compression was noted in the control group. Venous duplex waveforms were examined with arm abduction while the head was rotated either ipsilaterally or contralaterally from the abducted limb. In the musician group, head turned away from an abducted arm with resultant loss of bi-directional flow or diminution of normal phasicity occurred in 36% of subjects (*n =* 23), in contrast to 8% of the control subjects (*n =* 4, *p* < 0.03). When the head was turned toward an abducted arm, loss of bi-directional flow or diminution of normal phasicity occurred in 22% of musicians (*n =* 14) compared to 6% of the control subjects (*n =* 2). Overall abnormal venous waveform with arm abduction was noted in 56% of musicians compared to 13% of control subjects (*p =* 0.03). Arterial PPG tracings were analyzed with arm abduction while the head was positioned either toward or away from an abducted limb. Abnormal PPG results were defined by dampened or absent tracing, or provocative position to resting position velocity ratios greater than 2.0. Diminished or absent arterial PPG tracing with arm abduction was noted in 25% of musicians in contrast to 6% of control subjects (*p =* 0.04). Overall abnormal ultrasound or PPG test was found in 69% of musicians in contrast to 15% of control subjects (*p =* 0.03). Among these musicians, abnormal arterial PPG tracing or venous duplex waveform were detected in 56% of violists or violaists compared to 18% of cellists (*p =* 0.03). There is a statistical difference in arterial and venous flow anomalies based on PPG or ultrasound assessment in the left arm compared to the right arm in violinists and violaists (*p =* 0.04). There was no difference in the arm laterality with respected to abnormal PPG or waveforms in cellists. Subjects with positive provocative maneuvers were analyzed with abnormal ultrasound or PPG results in both musician and control group, and these results were shown in Table 5. In the musician group, there were 23 subjects (36%) with both positive provocative maneuver test and abnormal ultrasound results which was in contrast to three subjects (6%, *p =* 0.03) in the control group. When we analyzed the laterality of provocative maneuvers versus abnormal ultrasound or PPG results, there was no statistical difference between these variables in either the musician or control subjects. 

## 4. Discussion

High-performance musicians who perform instruments with repetitive physical motion can endure significant musculoskeletal strain and physical stress with time. Although multiple physical ailments related to nerve entrapment and joint disorders have been described in elite musicians [7,8,12,13,14,15], published reports of TOS in these musical instrumentalists remain scarce. The findings of our study are notable as it represents the first prospective evaluation demonstrating TOS is common among elite bowed string musicians, particularly violinists and violists, based on both physical examination as well as ultrasound and PPG assessment. 

Although TOS was first described in the early 19th century by Sir Astley Cooper, who treated a patient with a subclavian artery aneurysm caused by first rib compression [16], this condition has continued to pose a significant diagnostic challenge for clinicians due in part to its uncommon incidence, as well as a lack of a single diagnostic test to confirm this condition unequivocally. The Consortium for Outcomes Research and Education of Thoracic Outlet Syndrome recently proposed a preliminary set of diagnostic criteria for TOS [17]. A subsequent updated reporting standard for TOS was published by the Society for Vascular Surgery which include: symptoms of pathology at the thoracic outlet, symptoms of nerve compression, the absence of other pathology potentially explaining the symptoms, and a positive scalene muscle injection test [11]. While these guidelines are useful in differentiating various musculoskeletal ailments from TOS, there still remain many limitations. For instance, these standards recommend the use of scalene muscle injection for diagnostic evaluation, which is a highly specialized procedure that may not be readily available in many clinical practices. Many patients may be unwilling to undergo this invasive test for diagnostic evaluation. Additionally, these guidelines do not include ultrasound or PPG assessment for analyzing subclavian vessel compression. With these considerations in mind we, therefore, adopted more practical diagnostic criteria in our study by incorporating pertinent physical examinations, provocative maneuvers, and ultrasound assessment in our volunteer subjects. We also correlated positive provocative maneuvers with abnormal ultrasound findings for subclavian vessel compression to detect the incidence of TOS between the musician group and the control subjects.

Clinical studies utilizing duplex ultrasound to detect subclavian vessel compression in patients with TOS have reported varied results [1,2,18,19,20,21]. Orlando et al., reported their experience of 143 TOS patients who underwent bilateral preoperative duplex ultrasound prior to first rib resection and scalenectomy, and significant flow abnormality was defined as greater than 50% flow reduction with arm abduction. The authors found abnormal subclavian vessel compression in 49% of patients [20]. Demondion et al. used B-mode ultrasound to evaluate thoracic outlet arterial compression in 28 patients with TOS as well as 44 normal individuals, and reported six of the 44 volunteers (14.6%) had arterial stenosis greater than 70% when the arm is extended at 170° [18]. The finding of subclavian vessel compression in healthy individuals has also been reported by several researchers. A recent study from the University of Michigan examined bilateral duplex scans in 50 healthy volunteers and found abnormal duplex scans by either PPG waveforms or velocities in 60% of veins and 30% of arteries with significant variability with arm positioning [1]. The authors reported that dampened or absent PPG tracings are more reliable indicators of hemodynamically-significant vascular compression compared to flow velocities, as the PPG tracings are truly reflective of flow-restrictive changes occurring during positional changes. Colon et al., analyzed PPG finger tracing in 115 health subjects and noted that 44% of them experience severe arterial flow reduction when the arm is abducted in 120° position [2]. Similarly, Longley et al., evaluated 20 normal individuals along with 16 patients with TOS using Doppler ultrasound. They defined significant arterial compression as doubling of peak systolic velocity or complete flow cessation in hyperabduction, while venous abnormality as complete cessation of blood flow or loss of respiratory phasicity with arm hyperabduction. The authors found that 20% of the volunteers had abnormal arterial compression and 10% had significant venous compression with arm abduction [19]. Lastly, Rohrer et al., performed ultrasound evaluation in 46 volunteers including 19 major league baseball pitchers, 16 non-pitching major league players, and 11 non-athlete controls for thoracic outlet compression in the throwing position. They found 56% of these subjects had significant subclavian and axillary with blood pressure decrease of greater than 20 mm Hg. The authors concluded that repetitive upper extremity trauma of the throwing motion can the principle culprit of the arterial compression [21]. These studies all underscored a common observation that subclavian vessel compression can occur in healthy asymptomatic individuals. Furthermore, the degree of thoracic outlet vessel compression can be influenced by the provocative arm position, and results of subclavian vessel compression may vary based on the method of analysis. 

In our study, we defined abnormal scan as vessel compression of greater than 50% or reduced PPG waveforms with arm abduction. We found abnormal ultrasound scan by either PPG waveforms or velocities was detected in 69% of musicians, in contrast to 15% of volunteer subjects (Table 4). Those with positive provocative maneuvers with either EAST or ULTT in the musician and control groups were 44% and 6%, respectively (Table 3). Combining the findings of abnormal ultrasound scan and positive provocative maneuver tests, the diagnosis of TOS is made in 37% of musicians, in contrast to 6% of control subjects (Table 5). Among bowed string musicians, abnormal ultrasound scans were more commonly detected in violists and violists compared to cellists, which were 56% and 18% respectively. Although viola is a heavier instrument compared to violin, we did not find difference in subclavian vessel compression base on ultrasound assessment between violinists and violists. We postulate that, because violin and viola are similar string instruments which are positioned above the clavicle and stabilized by the left hand in an elevated position, this creates considerable physical strain to the thoracic outlet as well as the left arm. In contrast, a cello is a string instrument positioned on the ground which does not incur physical strain to the clavicle or requires the musician to maintain the musical instrument in an elevated position. 

Reports of musculoskeletal injures and nerve entrapment syndromes have been previously reported in bowed string musicians [7,15,22,23,24]. In a study analyzing 76 adolescent string musicians from the West Australian Youth Orchestras, 73.5% of the surveyed violinists reported upper extremity musculoskeletal symptoms with pain or paresthesia involving the shoulder, elbow, or wrist joint [25]. In two review articles which analyzed 342 and 117 musicians, respectively, researchers reported a high incidence of compression neuropathy involving the left arm among violinists, particularly left ulnar neuropathy at the elbow due to left forearm supination with wrist flexion while playing the instrument [6,8]. This elbow inward supination is necessary when playing the G or D strings, which are located on the left outer region of the strings. Another study has similarly documented ulnar nerve entrapment syndrome involving the left elbow among violinists, as evidenced by abnormal nerve conduction and electromyography studies [15]. Our study revealed a greater incidence of ultrasound abnormality and positive provocative maneuver tests in the left arm compared to the right arm among the violinists or violists, which underscores the physical stress endured by the left upper extremity in these musicians. Researchers have even coined the term “the droopy shoulder syndrome” to characterize an abnormal contour of the cervical and thoracic spines among violin players, which is attributed in part to chronic clavicle pressure exertion by the stringed instrument pressed against the left clavicle [26]. Using an infrared thermographic imaging, Clemente et al. documented left temporomandibular joint disorders in elite violists with muscle hyperactivity of the head and cervical muscles, and the authors postulated the temporomandibular disorder was due to strained shoulder and neck postures during long musical performances by bowed string instrumentalists [22]. Several reports have similarly described a high incidence of musculoskeletal ailments including functional dystonia involving upper extremities, due in part to abnormal posture and prolonged musculoskeletal strains among bowed string instrumentalists [7,13,15,23]. Other researchers have suggested that the neck position of violinists while playing may predispose them to cervical radiculopathy [12,27,28,29]. Taking into consideration these published reports, we speculate the physical strain experienced by high-performance bowed string musicians has contributed in part to the pathogenesis of various musculoskeletal disorders and nerve entrapment syndromes of the upper extremities, including TOS.

There are inevitably several weaknesses worth considering. Abnormal duplex ultrasound with subclavian vessel compression was not an infrequent finding as we detected this occurrence in 15% of our healthy volunteers. Consequently, critics may challenge the comparative value regarding the diagnostic sensitivity of TOS in these elite musicians when compared to the control subjects. Additionally, TOS is a clinical condition which encompasses a myriad of neurovascular compressive symptoms, and its diagnosis cannot be based on sonographic evidence of neurovascular compression alone. Although we utilized provocative maneuvers, such as the EAST and ULTT, to elicit symptoms of TOS, the reliability of these provocative physical tests combined with abnormal ultrasound assessment has not been validated with proven diagnostic sensitivity for TOS. In spite of these study limitations, our study underscored a causative relation between high-performance bowed string instrumentalists and TOS.

In conclusion, our study showed a high prevalence of neurovascular compression in elite stringed instrumentalists based on duplex ultrasound evaluation compared to control subjects. The abnormal ultrasound finding also correlates with provocative maneuvers. The finding of this study provides an important diagnostic insight regarding neuromuscular strain caused by chronic repetitive upper extremity physical motion in high-performance musicians. Clinicians should have a heightened level of diagnostic awareness for TOS when treating patients with thoracic outlet strain who are high-performance bowed string musical instrumentalists. 

## Figures and Tables

**Table 1:** Baseline demographic information and upper extremity activity.

Characteristics	Musician Group (*n =* 64)	Control Group (*n =* 52)	*p*-Value
Age, mean ± SD (years)	35 ± 9.3	28 ± 11.6	NS
Age, range (years)	21–53	19–48	
Gender			
Male	28 (44%)	25 (48%)	
Female	36 (56%)	27 (52%)	NS
Bowed string instrument played			
Violin	26 (41%)	N/A	
Viola	21 (33%)	N/A	
Cello	17 (27%)	N/A	
Duration of daily upper extremity repetitive activity (hour)	5.3 ± 2.4	0.6 ± 0.4	0.001

NS means non-significant.

**Table 2:** Comparison of physical examination between the musician and control groups.

Test Performed	Musician Group (*n =* 64)	Control Group (*n =* 52)	*p*-Value
Physical Examination (Localized Tenderness on Palpation)			
Scalene triangle tenderness (right)	6 (9%)	1 (2%)	
Scalene triangle tenderness (left)	16 (25%)	0	
Overall scalene triangle tenderness	22 (34%)	1 (2%)	0.03
Physical Examination (Localized Tenderness on Palpation)			
Subcoracoid space (right)	1 (2%)	1 (2%)	
Subcoracoid space (left)	2 (3%)	0	
Overall subcoracoid space tenderness	3 (5%)	1 (2%)	NS
Overall positive exam for localized tenderness	25 (39%)	2 (4%)	0.03

**Table 3:** Comparison of provocative maneuvers between the musician and control groups.

Test Performed	Musician Group (*n =* 64)	Control Group (*n =* 52)	*p*-Value
Provocative Maneuvers			
Positive EAST (right)	7 (11%)	1 (2%)	
Positive EAST (left)	11 (17%)	1 (2%)	
Overall Positive EAST	18 (28%)	2 (4%)	0.04
Provocative Maneuvers			
Positive ULTT (right)	5 (8%)	2 (4%)	
Positive ULTT (left)	12 (19%)	0	
Overall Positive ULTT	17 (27%)	2 (4%)	0.04
Overall positive provocative maneuvers	28 (44%)	3 (6%)	0.03

**Table 4:** Comparison of ultrasound and PPG evaluation between the musician and control groups.

Test Performed	Musician Group (*n =* 64)	Control Group (*n =* 52)	*p*-Value
Ultrasound Evaluation			
Subclavian vessel compression with arm abduction	30 (47%)	6 (12%)	0.04
Abnormal venous waveforms			
90° arm abduction, head turned contralateral	23 (36%)	4 (8%)	
90° arm abduction, head turned ipsilateral	14 (22%)	3 (6%)	
Overall abnormal venous waveform result	36 (56%)	7 (13%)	0.03
Abnormal arterial PPG tracing			
90° arm abduction, head turned contralateral	11 (17%)	2 (4%)	
90° arm abduction, head turned ipsilateral	7 (11%)	1 (2%)	
Overall abnormal arterial PPG tracing	16 (25%)	3 (6%)	0.04
Overall abnormal ultrasound or PPG test	44 (69%)	8 (15%)	0.03

**Table 5:** Assessment of positive provocative maneuvers with the presence of abnormal ultrasound or PPG results.

Diagnostic Study	Musician Group (*n =* 64)			Control Group (*n =* 52)		
	Positive EAST (*n =* 18)	Positive ULTT (*n =* 17)	Overall Positive Provocative Maneuvers (*n =* 28)	Positive EAST (*n =* 2)	Positive ULTT (*n =* 2)	Overall Positive Provocative Maneuvers (*n =* 3)
Subclavian vessel compression (>50%)	6 (9%)	4 (6%)	10 (36%)	1 (2%)	1 (2%)	2 (3%)
Abnormal venous waveforms	10 (16%)	7 (11%)	14 (50%)	1 (2%)	1 (2%)	2 (3%)
Abnormal arterial PPG tracing	7 (11%)	5 (8%)	11 (39%)	0	0	0
Overall abnormal ultrasound or PPG test	15 (23%)	14 (22%)	23 (36%) *	2 (3%)	2 (3%)	3 (6%) *

% = percentage of patient is calculated based on the total number of patients in the musician or control group respectively. * *p =* 0.03 when compared between the musician and control group.
